# RiboFACSeq: A new method for investigating metabolic and transport pathways in bacterial cells by combining a riboswitch-based sensor, fluorescence-activated cell sorting and next-generation sequencing

**DOI:** 10.1371/journal.pone.0188399

**Published:** 2017-12-06

**Authors:** Zohaib Ghazi, Shahrzad Jahanshahi, Yingfu Li

**Affiliations:** 1 Department of Biochemistry and Biomedical Sciences, McMaster University, Hamilton, Ontario, Canada; 2 School of Biomedical Engineering, McMaster University, Hamilton, Ontario, Canada; University of Cape Town, SOUTH AFRICA

## Abstract

The elucidation of the cellular processes involved in vitamin and cofactor biosynthesis is a challenging task. The conventional approaches to these investigations rely on the discovery and purification of the products (i.e proteins and metabolites) of a particular transport or biosynthetic pathway, prior to their subsequent analysis. However, the purification of low-abundance proteins or metabolites is a formidable undertaking that presents considerable technical challenges. As a solution, we present an alternative approach to such studies that circumvents the purification step. The proposed approach takes advantage of: (1) the molecular detection capabilities of a riboswitch-based sensor to detect the cellular levels of its cognate molecule, as a means to probe the integrity of the transport and biosynthetic pathways of the target molecule in cells, (2) the high-throughput screening ability of fluorescence-activated cell sorters to isolate cells in which only these specific pathways are disrupted, and (3) the ability of next-generation sequencing to quickly identify the genes of the FACS-sorted populations. This approach was named “RiboFACSeq”. Following their identification by RiboFACSeq, the role of these genes in the presumed pathway needs to be verified through appropriate functional assays. To demonstrate the utility of our approach, an adenosylcobalamin (AdoCbl)-responsive riboswitch-based sensor was used in this study to demonstrate that RiboFACSeq can be used to track and sort cells carrying genetic mutations in known AdoCbl transport and biosynthesis genes with desirable sensitivity and specificity. This method could potentially be used to elucidate any pathway of interest, as long as a suitable riboswitch-based sensor can be created. We believe that RiboFACSeq would be especially useful for the elucidation of biological pathways in which the proteins and/or their metabolites are present at very low physiological concentrations in cells, as is the case with vitamin and cofactor biosynthesis.

## Introduction

The products of vitamin and cofactor biosynthetic pathways are essential for the survival of all eukaryotic and bacterial microorganisms, whether they are produced by the organism or consumed from the environment. Since cofactors are usually catalytic in function [1], they are required in significantly lower quantities than the metabolites of central metabolic pathways. In addition, the biosynthesis of cofactors is usually strictly regulated and thus the enzymes involved in their assembly are similarly produced in scarce quantities [2]. This presents the conventional approaches to the study of vitamin and cofactor biosynthesis with significant technical challenges.

The elucidation of biosynthetic pathways classically relies on the coordinated use of multiple chemical and biochemical approaches, which typically include isotopic labeling, recombinant overexpression of proteins, and the complementary use of genetic screens [2,3]. However, they have certain drawbacks: (1) crucial to the success of these approaches is the serendipitous detection and isolation of auxotrophic mutants that lack enzymes involved in the pathway under investigation, especially when little to no information is available on the pathway [2]; (2) the comprehensive analysis of a pathway relies on the purification of target proteins and/or their metabolites through the conventional method of fractionation, which often requires multiple steps, specialized equipment (e.g. LC/GC-MS, HPLC, MS-MS, NMR, FT-IR) and trained personnel [4,5]. Although these approaches have been used successfully in the study of molecules involved in central metabolic pathways, the identification and separation of less abundant molecules such as those involved in vitamin and cofactor biosynthesis usually suffer from significant technical challenges; these include very low yields and large impurities, as well as consumption of large amounts of natural resources. Therefore, the traditional methods of investigation lack broad applicability, necessitating a more sensitive and comprehensive method for the systematic elucidation of bacterial transport and biosynthetic pathways.

We have previously reported a novel *in-vivo* based approach for studying the metabolism and transport of coenzyme B12, also known as adenosylcobalamin (AdoCbl; a biologically active form of vitamin B12), within living bacterial cells [6]. This approach involved the use of a riboswitch-based sensor. Specifically, an AdoCbl-responsive riboswitch fused to a reporter gene was successfully used to track the effects of varied growth conditions and genetic modifications on both the transport and biosynthesis of AdoCbl in *Escherichia coli* [6].

Having previously demonstrated the effectiveness of riboswitch-based sensors as intracellular tools for monitoring the physiologically relevant concentrations of their cognate molecules [6], we examined the prospect that these sensors could be used as a means to elucidate the pathway(s) involved in the production of these molecules. Any perturbations to the genes involved in their transport and biosynthesis should theoretically result in a change in fluorescence signal by the sensor in these cells relative to those with the intact versions of the genes. It seems thus plausible that these sensors could be exploited to identify the gene products that are essential for the transport and biosynthesis of a target molecule by screening a library of random mutants for those that exhibit a change in fluorescence signal. As a proof of concept, we investigated AdoCbl transport and biosynthesis in *E*. *coli* by using an AdoCbl-responsive riboswitch-based sensor.

Physiologically, *E*. *coli* lacks the complete set of genes required for de novo biosynthesis of AdoCbl [7–9]; however, it encodes the transport proteins and metabolic enzymes required for the import and salvage synthesis of AdoCbl from exogenous corrinoids (molecules containing the cobalt-containing cyclic tetrapyrole known as the corrin ring), such as cobinamide (Cbi) and vitamin B12 (VB_12_: cyanocobalamin, CNCbl). The salvaging of exogenous corrinoids (e.g. Cbi) begins with their transport into the cell by the TonB-dependent BtuBFCD transport system (panel B in S1 Fig) [10–13]. The BtuB protein delivers the extracellular corrinoid to the periplasmic corrinoid-binding protein BtuF [14–16]. The energy required for this function is acquired through interactions with an inner membrane protein complex comprised of proteins TonB, ExbB and ExbD [17–20]. Once bound with the corrinoid, BtuF delivers the molecule to the inner-membrane ABC transporter BtuCD [14,21,22], which translocates the molecule into the cytoplasm using ATP hydrolysis.

Upon entry into the cell (panel C in S1 Fig), the salvaging of the incomplete corrinoid (e.g. Cbi) begins with the attachment of the upper axial ligand. Cbi is adenosylated by the ATP:corrinoid adenosyltransferase enzyme BtuR [23–25], which yields AdoCbi. Next, AdoCbi is transformed into AdoCbi-GDP via intermediate AdoCbl-P in a two-step process catalyzed by the bifunctional enzyme CobU [26,27]. Concurrently, a limited range of lower ligand bases can be activated for attachment to the incomplete corrinoid by the phosphoribosyl transferase enzyme CobT [26,28–31]. Nevertheless, in the presence of DMB and nicotinate mononucleotide (NaMN), the ADP-ribosyltransferase activity of CobT yields α-ribazole-5’-phosphate (α-RP), the intermediate in the synthesis of the nucleotide loop of cobalamin. Then, the condensation of AdoCbi-GDP and α-RP is catalyzed by the AdoCbl-5’-P synthase enzyme CobS [32–34]. Finally, the AdoCbl-P phosphatase enzyme CobC catalyzes the removal of the 5’-O-P from AdoCbl-5’-P to yield the end product of the pathway [33,35,36], namely AdoCbl.

**Fig 1:**
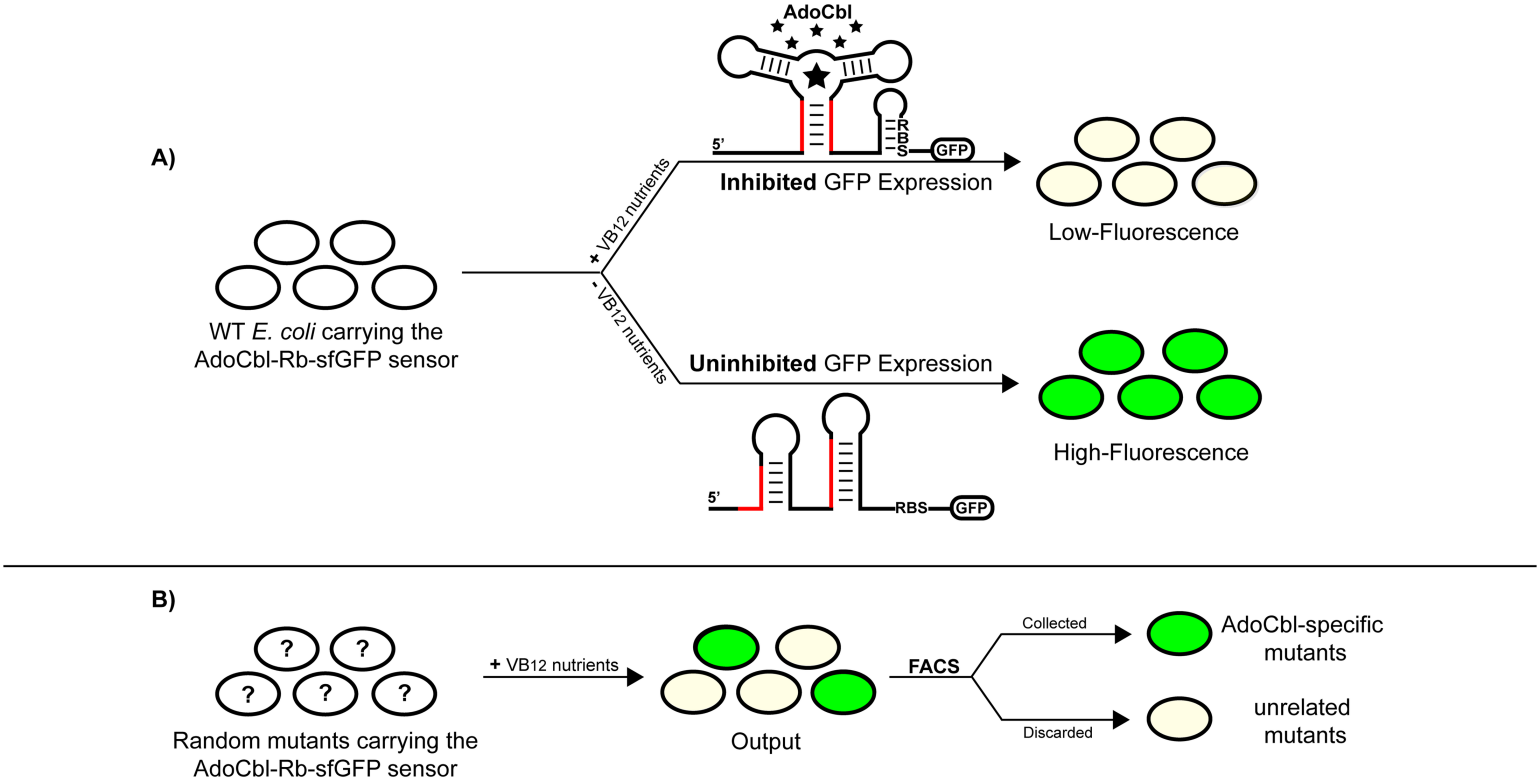
Schematic of the riboswitch sensor mediated fluorescence activated cell sorting (RiboFACS) method. (A) The detection of intracellular adenosylcobalamin (AdoCbl) by the AdoCbl-responsive riboswitch-based sensor (AdoCbl-Rb-[sfGFP] sensor) in *E*. *coli* cells. Since *E*. *coli* lacks the complete de novo pathway for AdoCbl biosynthesis, the AdoCbl-Rb-sfGFP sensor can be used to detect the presence or absence of intracellular AdoCbl in cells cultured with or without VB12 nutrients. At high concentrations of cellular AdoCbl (stars), the riboswitch region of the sensor could adopt the ligand-bound structure, which sequesters the ribosome-binding site (RBS) and inhibits downstream *gfp* expression (i.e. host cell exhibits low-fluorescence). Conversely, at very low or undetectable concentrations of cellular AdoCbl, the same region remains in the ligand-unbound structure, which permits downstream *gfp* expression (i.e. host cell exhibits high-fluorescence). Red-highlighted segments represent complementary regions of nucleic acids. (B) The isolation and identification of ‘AdoCbl-specific’ mutants defective in AdoCbl transport or biosynthesis by screening a pool of randomly mutagenized cells carrying the aforementioned sensor. Theoretically, a library of randomly mutagenized cells may include (1) mutants with disrupted AdoCbl transport or biosynthetic processes, which exhibit high fluorescence despite the presence of VB12 nutrients; or (2) those without such disruptions (i.e. unrelated mutants), which exhibit low-fluorescence under these circumstances. Thus, we could separate AdoCbl-specific mutants from unrelated mutants by using fluorescence-activated cell sorting (FACS) as a means to identify the genes that would normally be involved in the transport and biosynthesis of AdoCbl in *E*. *coli*.

To understand the utility of riboswitch-based sensors, in particular the AdoCbl-responsive riboswitch-based sensor in this study, as part of our RiboFACSeq approach, let us first consider the behavior of the sensor in wild-type (WT) *E*. *coli* cells (Fig 1A). When WT cells are grown in media supplemented with ‘VB12 nutrients’ (e.g. CNCbl or both Cbi & DMB), they can accumulate high concentrations of intracellular AdoCbl. Consequently, the riboswitch region of the sensor adopts the ligand-bound structure, which sequesters the ribosome-binding site (RBS) and inhibits downstream reporter gene expression (e.g. host cell exhibits low fluorescence) [6]. Alternatively, in the absence of VB12 nutrients, the same region remains in the ligand-unbound structure, which permits downstream reporter gene expression (e.g. host cell exhibits high fluorescence) [6].

Similarly, as shown in Fig 1B, if a cell carries a mutation that disrupts its ability to transport or synthesize AdoCbl (herein referred to as an ‘AdoCbl-specific mutant’), the riboswitch region of the sensor will remain in the ligand-unbound structure and permit downstream reporter gene expression (e.g. host cell exhibits high-fluorescence), despite the presence of VB12 nutrients in the media.

As such, we predict that a library of random mutants that carry the AdoCbl-responsive riboswitch-based sensor could be sorted (via fluorescence-activated cell sorting [FACS]) for AdoCbl-specific mutants by first growing them with VB12 nutrients and then selecting only those cells that exhibit high levels of reporter fluorescence (Fig 1B). Subsequent high-throughput sequencing (via next-generation sequencing) of the FACS-sorted cell fractions should permit quick identification of the genes potentially involved in the transport and biosynthesis of AdoCbl in *E*. *coli*. The role of the identified genes in the biosynthetic and/or transport pathways under investigation can subsequently be verified through appropriate functional assays.

As a step towards this ultimate goal, in this study, we demonstrate the ability of our approach to isolate AdoCbl-specific mutants from artificial cell mixture samples, which contain these mutants as well as those unrelated to AdoCbl transport and biosynthesis, and WT cells. In addition, the sensitivity and specificity with which AdoCbl-specific mutants are isolated from these samples are investigated.

Herein, we demonstrate that a metabolite-responsive riboswitch-based sensor combined with fluorescence-activated cell sorting and next-generation sequencing (dubbed “RiboFACSeq”) is an effective method for the systematic elucidation of the transport and biosynthetic pathways of a target molecule within living bacterial cells.

## Materials and methods

### Bacterial strains and genomic barcoding

*Escherichia coli* DH5αZ1 was employed for plasmid cloning. The assays, however, were conducted using the following select strains from the ‘Keio’ *E*. *coli* single-gene deletion library [37]: wild-type (WT), *ΔbtuB*, *ΔcobU*, *ΔcobS*, *ΔcobT*, *ΔcobC*, *ΔexbB*, and *ΔcarB*, which were derived from the parental strain BW25113 [*Δ(araD-araB)567 Δ(rhaD-rhaB)568 ΔlacZ4787 (*::*rrnB-3) hsdR514 rph-1*] [38]. To encode genetic barcodes, these strains were modified as follows. Initially, the kanamycin-resistance cassettes present within most of these strains were removed using FLP recombinase, as described by Datsenko and Wanner [39]; however, this step was omitted for strains *ΔbtuB* and *ΔcobT*, as they were selected using a Cm^R^ cassette that lacked the FRT-flanking regions [6]. Next, the (Km^S^) strains were individually targeted for gene disruption at the common genomic locus *ΔlacZ* with slight modifications to the described protocol [37,39]; specifically, for each strain, the N-terminal deletion primer was altered by the incorporation of an identifiable “barcode”–i.e. a unique 3-nt sequence—that was placed between the target gene sequence and the downstream FRT region. Finally, through lambda red-mediated recombination, genetically-barcoded clones of each strain were isolated and PCR-verified.

### Molecular cloning

The AdoCbl-responsive riboswitch-based sensor was constructed, as previously described [6], in a stepwise fashion using well-established protocols for cloning, restriction digestion, ligation, and transformation, as defined by respective manufacturer’s.

First, the AdoCbl riboswitch (-310 to +210 *btuB*) was amplified from *E*. *coli* K-12 strain *BW25113* genomic DNA using primers btuB-FOR-KpnI (5’- CTAGTAGGGTACCAGATCTTGATGAATTCCTATTTGTG) and btuB-REV-XFP (5’-GGTCAATGATGTGCTGCGCCGTCTTCCG ATGGTGAGCAAGGGCGAG). Next, the superfolder-GFP fluorescent protein reporter gene [40] was amplified from a plasmid EGFP-C1 by using primers btubXFP-FOR (5’-GTGCTGCGCCGTCTTCCGATGGTGAGCAAGGGCGAGGAG) and FP-REV-XbaI (5’-GCATGGACGAGCTGTACAAGTAATCTAGAGCATCAT). Through crossover-PCR, the ~520-nt AdoCbl riboswitch fragment was fused in-frame to the ~740-nt sfGFP reporter gene fragment using primers btuB-FOR-KpnI and FP-REV-XbaI. Lastly, the resulting ~1.2-kb DNA fragment was cloned into the KpnI and XbaI site’s of a modified pBAD18 vector, which had its arabinose-responsive elements removed, to form the AdoCbl-responsive riboswitch-based sensor that regulates the expression of a sfGFP fluorescent reporter protein (abbrev as AdoCbl-Rb-sfGFP sensor).

Each genetically-barcoded strain was transformed with the AdoCbl-Rb-sfGFP sensor using electroporation, and their positive clones were selected by spreading cultures onto Luria-Bertani (LB) agar plates supplemented with 100-μg/mL ampicillin.

### Media, low-throughput fluorescence assays and signal normalization

*E*. *coli* K-12 cells were cultured in a rich, chemically defined medium (RDM; described in [6]) supplemented with 75-μg/mL ampicillin and, wherever specified, 500 nM of either vitamin B12 (supplied as cyanocobalamin, CNCbl) or its precursors cobinamide (supplied as cobinamide dicyanide, Cbi) and 5,6-dimethylbenzimidazole (DMB). Cell cultures were incubated at 37°C with shaking at 250 rpm. For fluorescence assays, initially, starter cultures in RDM were set using colonies picked from LB-agar plates representing each strain, and they were grown overnight (~16–18 hrs). The overnight cultures were then used to inoculate assay cultures (at 1:1000 dilution) in RDM supplemented with the following compounds: (1) neither CNCbl nor Cbi nor DMB (i.e. “no B12”); (2) CNCbl; or (3) Cbi and DMB; (all purchased from Sigma). These cultures were grown until they reached ~mid-late log phase. Prior to reading fluorescence, ~1.2 ml of each sample was collected (5000 rpm for 5 min) and washed, thrice, using an equal volume of 1xPBS, before resuspending to a final volume of 1 ml of 1xPBS. The TECAN M1000 (Safire) plate-reader was used to read sfGFP fluorescence (488/509 nm). To quantify autofluorescence, each of the steps mentioned above was repeated using untransformed cells grown in RDM devoid of antibiotics. For signal normalization, raw reporter activities were corrected for growth differences (using OD600), log-transformed, and plotted as bar graphs for visual analysis. The magnitude of fluorescence-inhibition in response to the specified vitamin B12 supplement(s) was calculated by dividing the OD600-normalized fluorescence value in the “no B12” condition by that in the presence of the indicated nutrient(s). In all cases, each sample was assayed in triplicate, and its standard deviation was reported as error bars.

### Preparation of artificial cell mixture samples

To assess RiboFACS, several artificial cell mixture samples were prepared, as follows. Initially, the cell concentrations of each strain at ~mid-late log phase were estimated by plating serial dilutions of each sample onto LB-agar plates supplemented with 100-μg/mL ampicillin. Provided with these estimates, sample tubes containing indicated amounts of each specified strain were combined as described in the paper. The concentration of each sample was limited to no higher than ~10^6^ cells/mL. In all cases, to stain dead cells, sample tubes were spiked with propidium iodide to a final concentration of 30 μM. Finally, immediately prior to their flow cytometric evaluation, each sample tube was passed through a 35-μm cell-strainer cap.

### System, software, and laser parameters

The BD FACSAria^™^ III flow cytometry system, operating on the FACSDiva^™^ v6.1.3 software (BD Biosciences), was used to sort and document cells; however, data analysis was performed on the FlowJo for Mac version 10.0 analysis software (TreeStar). A 488-nm laser was employed to detect light scatter and fluorescence measurements of individual cells, recording data under the following parameter’s: Forward-scatter light (FSC): 280V, linear scale; Side-scatter light (SSC): 290V, 488 ±10 nm bandpass filter (BP), linear scale; FITC-A (i.e. GFP fluorescence): 390V, 530 ±30 nm BP, logarithmic scale; PE-Texas Red-A (i.e. propidium iodide levels): 504V, 616 ±23 nm BP, logarithmic scale; A cutoff threshold of FSC: 1010 & SSC: 500 was applied to reduce and remove non-cell related instrumental noise and background. Data corresponding to the GFP and PI fluorescence channels were obtained on a logarithmic scale; however, they were stored and exported on a linear scale. The rate of acquisition and cell sorting ranged between 1500–3000 events/sec, depending on the requirements of the particular experiment.

### Gating parameters

Cell cultures were subjected to the following scheme. First, a selection gate was applied around the bi-exponential contour dot plot resulting from the distribution of cells in light scatter based on size (forward scatter; FSC) and intracellular composition (side scatter; SSC) [41]–i.e., events that resemble *E*. *coli* cells were selected. Second, to distinguish alive from dead cells, a gate was applied around the bi-exponential contour dot plot of PI-negative events, which represent the fraction of viable cells. Third, a doublet discrimination method was used to probe and exclude events that could represent more than 1 cell by deconvoluting each event’s pulse-signal [41,42]–i.e., SSC and FSC were used, hierarchically, to disregard non-single-cell events by placing appropriate gate’s around the log-linear contour dot plots of SSC and FSC’s—height vs.—width, respectively. Finally, the distribution of fluorescence intensity among the cells of an assayed sample was represented as a contour dot plot and fluorescence histogram.

### Read counts

Sequencing reads obtained from Illumina MiSeq were processed for adapter sequences using CutAdapt version 1.14 (https://github.com/marcelm/cutadapt) [43] and screened for genetic barcodes using BBDuk from the BBMap package version 37.36 (https://sourceforge.net/projects/bbmap/) [44]. First, CutAdapt processing parameters were set to remove both 5’ (CAGCTTATCATCGGAGCTCC) and 3’ (CCTATACTTTCTAGAGAATA) adapter sequences (i.e. “linked adapters”) error-tolerantly from each read pair, discarding those that lacked a 5’ read match. Subsequently, trimmed reads were shortened to a final length of 38 nucleotides (by removing bases from the 3’ end). Finally, the processed read pairs were screened for specific genetic barcodes (S1 Table) using BBDuk, which was set to match only forward k-mers (rcomp = f) using a k-mer size of 9.

## Results and discussion

### Construction and functional validation of the AdoCbl-responsive riboswitch-based sensor

To select a reporter gene for our AdoCbl-responsive riboswitch-based sensor, the excitation/emission wavelengths of a range of fluorescent proteins were considered with respect to their compatibility with the available laser and filter parameters in the intended flow cytometry instrument; superfolder-GFP (sfGFP) was chosen as the reporter protein for the sensor construct. As previously described [6], the *sfGFP* reporter gene was fused downstream and in-frame to the AdoCbl-responsive riboswitch from *E*. *coli* [45], and cloned into modified vectors derived from the pBAD series of plasmids [46]. Herein, this sensor will be referred to as AdoCbl-Rb-sfGFP sensor.

**Fig 2:**
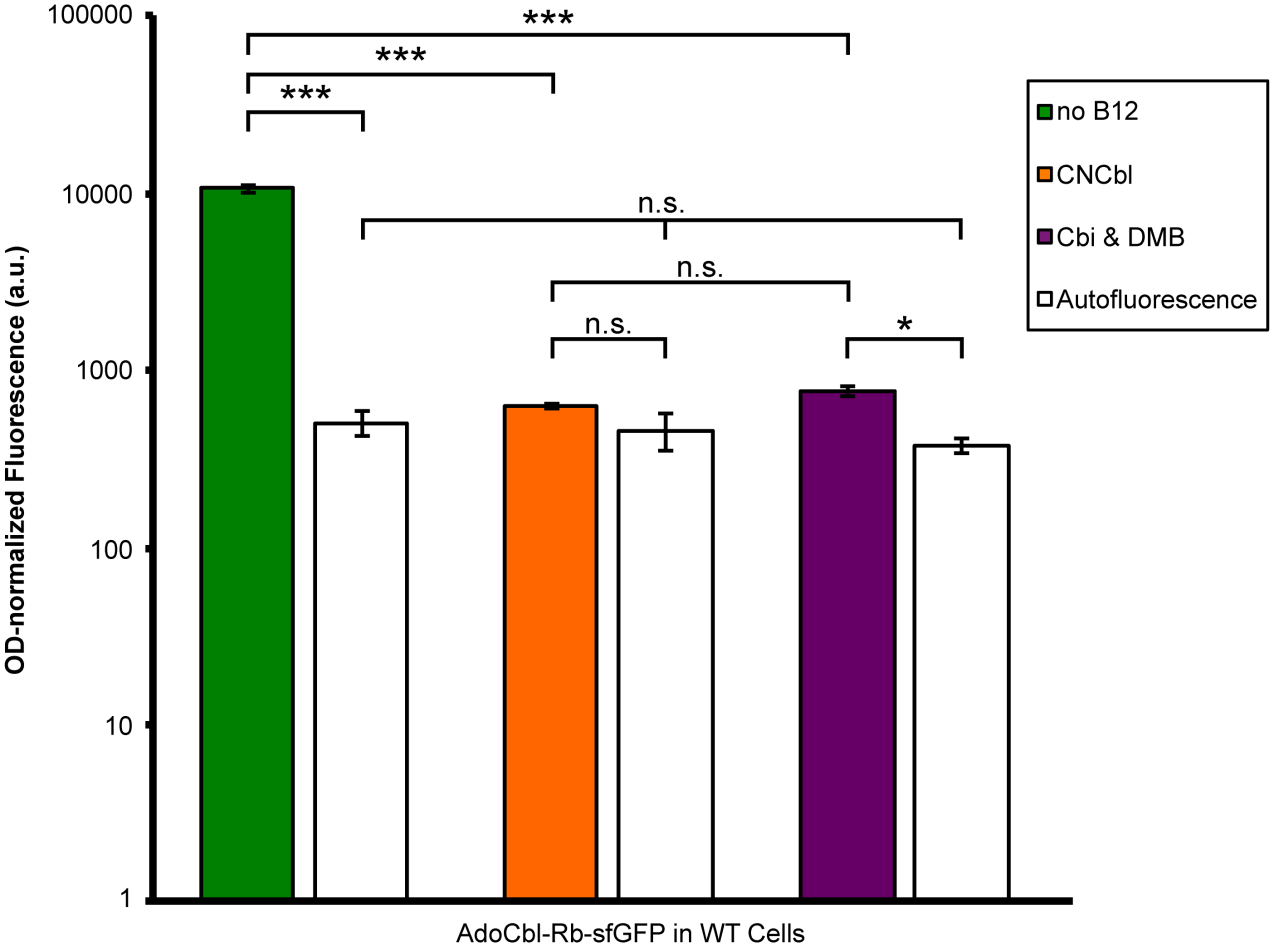
Probing adenosylcobalamin (AdoCbl) transport and biosynthesis in wild-type *E*. *coli* using an AdoCbl-responsive riboswitch-based sensor. Parallel cultures were grown in media supplemented with the following compounds: (i) neither cyanocobalamin (CNCbl) nor cobinamide (Cbi) nor 5,6-dimethylbenzimidazole (DMB)–i.e. “no B12” (green); (ii) CNCbl (orange); and (iii) Cbi & DMB (purple). Similarly, WT cells lacking the sensor were also grown in each of the indicated media conditions to measure autofluorescence (white). Respective reporter activities were measured, corrected for growth differences (OD600-normalized), and the data were log-transformed. Each bar represents the average of three biological replicates with errors as standard deviations. A two-way ANOVA (with Bonferroni corrections) was run to determine the statistically significant differences between the samples (*, p-value < 0.05; **, p-value < 0.01; ***, p-value < 0.001; n.s., not significant).

To validate that the AdoCbl-Rb-sfGFP sensor can detect the transport and biosynthesis of AdoCbl in *E*. *coli*, the fluorescence response of the sensor to a variety of VB12 nutrient conditions was examined in WT *E*. *coli BW25113* strain carrying this construct (Fig 2). Specifically, using a fluorimetric plate reader, fluorescence activity was measured from cells grown in media containing the following supplements: (i) no CNCbl nor Cbi nor DMB—i.e. “no B12”; (ii) CNCbl; or (iii) Cbi & DMB. As shown in Fig 2, under these nutrient conditions, the sensor exhibited the expected fluorescence signals in WT cells: uninhibited fluorescence when grown in the absence of VB12 nutrients and inhibited fluorescence when grown in their presence. This is in agreement with previous observations [6].

Having validated the function of the sensor to detect intracellular AdoCbl, we predicted it would be possible to identify AdoCbl-specific mutants based on their fluorescence intensities; unlike WT cells, these mutants are unable to synthesize cellular AdoCbl when provided with exogenous sources of VB12 nutrients. To confirm this prediction, WT and the following *E*. *coli* single-gene knockout strains were selected from the Keio collection for further investigation [37]: those with disruptions in the synthesis of AdoCbl [7,36], namely *ΔcobU*, *ΔcobS*, *ΔcobT*, and *ΔcobC*; one with a disruption in the outer membrane transport of VB12 and its derivatives [10,47], namely *ΔbtuB*; one with a disruption in the energy transduction complex required for the uptake of VB12 [48,49,19,20], namely *ΔexbB*; and finally, a mutant control, namely *ΔcarB*, which is unrelated to the transport and biosynthesis of AdoCbl [50]. These strains were individually grown in the absence or presence of CNCbl or Cbi & DMB, and their reporter activities were measured using a fluorimetric plate reader (S2 Fig). For each strain, the percent change/reduction in fluorescence intensities for cells that were grown in the presence of CNCbl or precursors Cbi & DMB were displayed relative to fluorescence intensities in the absence of these supplements from the media (i.e. “no B12”); notably, “no B12” signals for each strain were set to 100% of the achievable intensity. A significant reduction in fluorescence intensities would indicate the presence of cellular AdoCbl, whereas an insignificant reduction would suggest no detectable AdoCbl in the cytoplasm, as a result of either no VB12 nutrients in the media or the inability of host cells to transport and synthesize AdoCbl from the media. In addition, for the same data set, the fold-differences in reporter activities (i.e. fluorescence) for each strain were also calculated by dividing the fluorescence measured in host cells grown in the absence to that in the presence of the indicated nutrient(s) (S3 Fig). A ratio of ‘1’ would suggest no detectable AdoCbl in the cytoplasm, as a result of either no VB12 nutrients in the media or the inability of host cells to transport and synthesize AdoCbl from the media, whereas higher values would indicate the presence of cellular AdoCbl.

**Fig 3:**
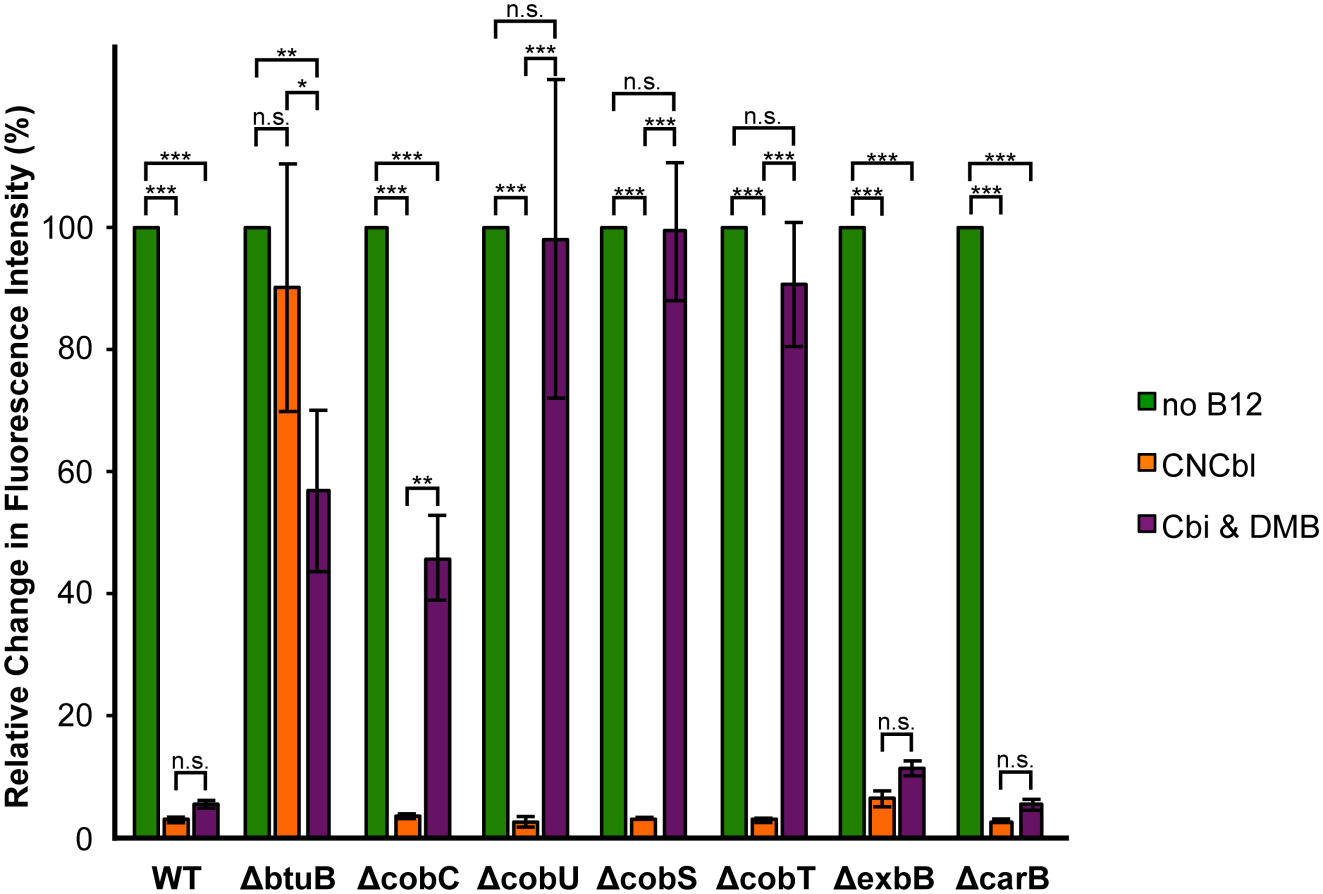
The adenosylcobalamin (AdoCbl)-responsive riboswitch-based sensor can distinguish both WT and an unrelated mutant from those with disrupted AdoCbl transport or biosynthesis in *E*. *coli*. The ability of each strain to transport and synthesize AdoCbl was examined by measuring the reporter activities of cells grown in media supplemented with the following compounds: (i) no cyanocobalamin (CNCbl) nor cobinamide (Cbi) nor 5,6-dimethylbenzimidazole (DMB); (ii) CNCbl; and (iii) Cbi & DMB. Subsequently, for each strain, the raw reporter activities were corrected for growth differences (OD600-normalized), and then used to determine the relative change in fluorescence intensity in response to the indicated compound(s) when compared to their absence. In other words, for each strain, the relative change in fluorescence intensity was determined by calculating the percent change in fluorescence intensity in response to CNCbl (orange) or both Cbi & DMB (purple), respectively, when “no B12” (green) was set to 100% of the achievable signal. A significant reduction in fluorescence intensities relative to “no B12” would indicate the presence of cellular AdoCbl, whereas an insignificant reduction would suggest no detectable AdoCbl in the cytoplasm, as a result of either no VB12 nutrients in the media or the inability of host cells to transport and synthesize AdoCbl from the media. Each bar represents the average of three biological replicates with errors as standard deviations. A two-way ANOVA (with Bonferroni corrections) was run to determine the statistically significant differences between the samples (*, p-value < 0.05; **, p-value < 0.01; ***, p-value < 0.001; n.s., not significant).

As shown in Fig 3, the AdoCbl-Rb-sfGFP sensor can be used to distinguish both WT and an unrelated mutant from those with disrupted AdoCbl transport or biosynthesis in *E*. *coli*. The fluorescence profiles of the assayed strains revealed several key patterns, as described below.

First, as expected, the controls (WT and *ΔcarB*) displayed nearly identical fluorescence behavior in response to the presence of CNCbl or precursors Cbi & DMB (Fig 3). Since the AdoCbl transport and biosynthetic pathways remained intact in these strains, the normal transport and biosynthesis of AdoCbl from the media facilitated the efficient inhibition of sfGFP fluorescence expression, and hence reduced fluorescence by the sensor. Interestingly, the relative changes in fluorescence intensities in these strains were stronger when the media contained CNCbl (~>97% reduction) rather than Cbi & DMB (~94% reduction). Although statistically insignificant, these differences could be physiologically meaningful and a reflection of the additional steps, time, and energy required for the metabolic transformation of precursors Cbi & DMB to AdoCbl, as that of CNCbl.

Secondly, the relative changes in fluorescence intensities of the AdoCbl-specific mutants correlated well with their known functionality in AdoCbl transport and biosynthesis (Fig 3). The VB12 outer membrane transport mutant *ΔbtuB* demonstrated a statistically insignificant reduction in relative fluorescence intensity in response to CNCbl, as expected, but displayed a statistically significant reduction in response to Cbi & DMB, presumably due to a low level of diffusion. This is consistent with previous reports that found mutants defective in B12 utilization due to a mutation in *btuB* could survive in vitamin B12-requiring conditions when supplied at a sufficiently high concentration [10,47,51]. On the other hand, the biosynthetic mutants of AdoCbl (*ΔcobU*, *ΔcobS*, *ΔcobT* and *ΔcobC*) exhibited statistically significant reductions in their relative fluorescence intensities compared to their controls, but only in response to CNCbl. In the presence of Cbi & DMB, however, most of these mutants exhibited statistically insignificant reductions in their relative fluorescence intensities. These findings were not surprising, since these cells contained genetic mutations that disrupted their ability to synthesize AdoCbl from precursors Cbi & DMB, but retained the ability to convert CNCbl to AdoCbl. Overall, the AdoCbl-Rb-sfGFP sensor demonstrated the ability to help identify and differentiate between AdoCbl transport and biosynthetic mutants.

Although most of the AdoCbl-specific mutants behaved as expected, the one exception was for *ΔcobC*, which displayed a statistically significant reduction (~54%) in its relative fluorescence intensity when grown with Cbi & DMB. As previously suggested [6], the function of CobC, a phosphatase involved in dephosphorylation of AdoCbl-P [36], may be partially compensated by a putative nonspecific phosphatase, which could explain the observed reduction in fluorescence. This theory is supported by circumstantial evidence indicating that the *ΔcobC* strain from *S*. *typhimurium* accumulates minor amounts of dephosphorylated AdoCbl [36]. Another possibility is that the sensor is able to also detect CobC’s substrate, phosphorylated AdoCbl. Recently [52], it has been shown that the corrin moiety of AdoCbl is the principle structural determinant for inducing the conformational switch of the *E*. *coli btuB* riboswitch. Interestingly, neither of the axially bound ligands was found to be required for stimulating the conformational change of the *btuB* RNA, but they were shown to enhance the affinity of corrinoid binding to the riboswitch. Only the removal of both axial ligands lowered the affinity so much that no binding occurred. These findings strongly support our claim that the AdoCbl-Rb-sfGFP sensor could interact with AdoCbl-P, as it has a corrin ring moiety, an upper axial ligand, and a lower axial ligand, which according to the study should be sufficient for interaction with the riboswitch to induce its conformational switch. However, the phosphorylated lower axial ligand may result in a lowered binding affinity to the AdoCbl-responsive riboswitch when compared to dephosphorylated AdoCbl.

Lastly, negligible defects in AdoCbl transport and biosynthesis were observed in cells carrying the *ΔexbB* mutation. Normally, ExbB is involved in an energy transduction complex that is required for the uptake of VB12 [53]. As such, it was reasonable to expect that the genetic deletion of protein ExbB would produce a phenotype resembling those of AdoCbl-specific mutants; instead, *ΔexbB* mutants exhibited statistically significant reductions in their relative fluorescence intensities in response to the VB12 nutrients (AdoCbl, ~94% reduction; Cbi & DMB, ~89% reduction), comparable to those observed in the controls (WT and *ΔcarB*). The results observed can be explained by the fact that the function of ExbB has been shown to be replaced by homologous proteins in *E*. *coli* [19,54,55].

Altogether, these findings demonstrate that the AdoCbl-Rb-sfGFP sensor can be used to probe the integrity of the AdoCbl transport and biosynthetic processes in host cells that carry them. In fact, the fluorescence profiles of host-strains make it possible to identify and distinguish between AdoCbl transport and biosynthetic mutants. Finally, this sensor may also be able to identify auxiliary or accessory genes related to AdoCbl transport and biosynthesis, as suggested by the fluorescence inhibition profile that is unique to *ΔexbB* relative to cells either with intact AdoCbl transport and biosynthetic pathways or with disruptions to these pathways that are indispensable.

As an extension of the aforementioned applications, in the following sections, we combine the use of this sensor with FACS as a means for the high-throughput separation of AdoCbl-specific mutants from artificial cell mixture samples based on their fluorescence intensities. First, an appropriate gating strategy for the isolation of AdoCbl-specific mutants is established by comparing the fluorescence activities of the representative strains with intact or disrupted AdoCbl transport pathways. Subsequently, both the sensitivity and specificity with which the RiboFACS-based method isolates the AdoCbl-specific mutants from artificial cell mixture samples are assessed. In both cases, sequencing is performed on the FACS-sorted cell fractions for the identification of the mutated genes.

### Establishing the flow-cytometric parameters for the isolation of AdoCbl-specific mutants from cell mixtures

To establish an appropriate set of criteria (a “gating strategy”) for the FACS-based isolation of AdoCbl-specific mutants from artificial cell mixture samples, the fluorescence activities of the representative strains with intact and disrupted AdoCbl transport pathways (i.e. WT and *ΔbtuB*, respectively) were analyzed using a flow cytometer. Prior to flow cytometry, both WT and *ΔbtuB* cultures, carrying the AdoCbl-Rb-sfGFP sensor, were independently grown in media supplemented with CNCbl, which inhibits fluorescence expression in WT but not *ΔbtuB* cells (see Fig 3). The fluorescence data on ~30,000 cells from each culture were collected using the FACSAriaIII flow cytometry system, and flow analysis was performed using the Flow Jo v10.0 analysis software, as described (see gating parameters).

**Fig 4:**
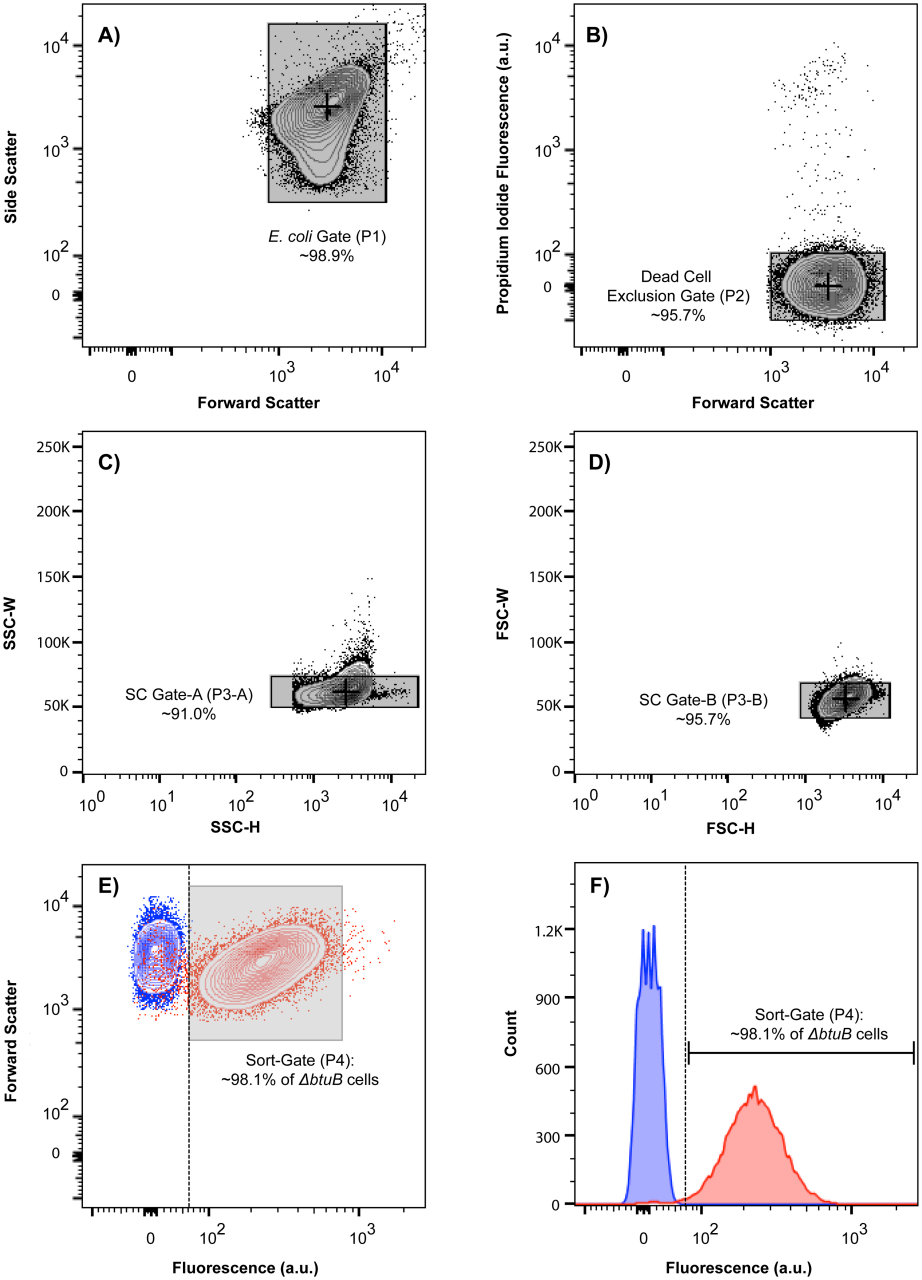
A gating strategy for the detection and isolation of mutants with defects in the transport or biosynthesis of adenosylcobalamin (AdoCbl) in *E*. *coli*. The reporter activities in representative strains with intact and disrupted AdoCbl transport pathways (i.e. WT and *ΔbtuB*, respectively) were examined using a flow cytometer. Initially, these strains were independently grown in media supplemented with CNCbl, which has previously been shown to inhibit reporter gene expression in WT but not *ΔbtuB* cells (see Fig 3). Subsequently, fluorescence data for ~30,000 cells from each matured culture was collected using the FACSAriaIII flow cytometry system, whereas analysis was performed using the Flow Jo v10.0 analysis software, as follows: (A) Selection gate for *E*. *coli* cells, P1 [FSC-A vs SSC-A]. *E*. *coli* cells were identified based on estimates of both cell size and internal complexity. (B) Dead-cell exclusion gate, P2 [FSC-A vs PI]. *E*. *coli* cells were examined for their ability to uptake propidium iodide [PI] and only PI-negative cells were selected. (C & D) Single-cell gates, P3-A [SSC-H vs–width] and P3-B [FSC-H vs—width], respectively. Living cells were subjected to these gates to remove any clumps or doublets. (E & F) Distribution of fluorescence intensity among the cells obtained from the WT (blue) and *ΔbtuB* (red) populations, as represented by a contour dot plot and a superimposed fluorescence histogram, respectively. The data were analyzed to define a sort-gate (P4; grey box) such that it prioritized the exclusion of WT cells at the cost of losing a variable amount of *ΔbtuB* cells. The dashed lines denote the lower-limit fluorescence intensity threshold of sort-gate P4.

As shown in Fig 4 (panels E & F), there is a notable difference in the mean fluorescence intensity (MFI) between the *ΔbtuB* (red) and WT (blue) populations. Specifically, under the specified assay conditions, the MFI value of the *ΔbtuB* population (238 a.u.) was approximately 34-times higher than the WT population (7 a.u.). As such, it was reasonable to assume that we could successfully separate a mixture of these strains by having the minimum fluorescence intensity threshold of a sort gate placed immediately after the fluorescence histogram of the WT population. Such a gating strategy would theoretically result in the isolation of ~98.5% of the cells in an isogenic *ΔbtuB* population (red), while discriminating against the isolation of nearly all (>~99.99%) of the cells in an isogenic WT population (blue). Although increasing this threshold value could achieve greater *ΔbtuB* cell purity, the ultimate goal of this study was the purification of AdoCbl-specific mutants that may not exhibit a comparable magnitude of fluorescence expression to that observed in the *ΔbtuB* population. By keeping the sort gate at the described position, we would prevent the loss of such AdoCbl-specific mutants. As such, this gating strategy was deemed appropriate for the isolation of AdoCbl-specific mutants from artificial cell mixtures, and both the sensitivity and specificity with which this goal was achieved remained to be investigated.

### Sensitivity assessment

**Fig 5:**
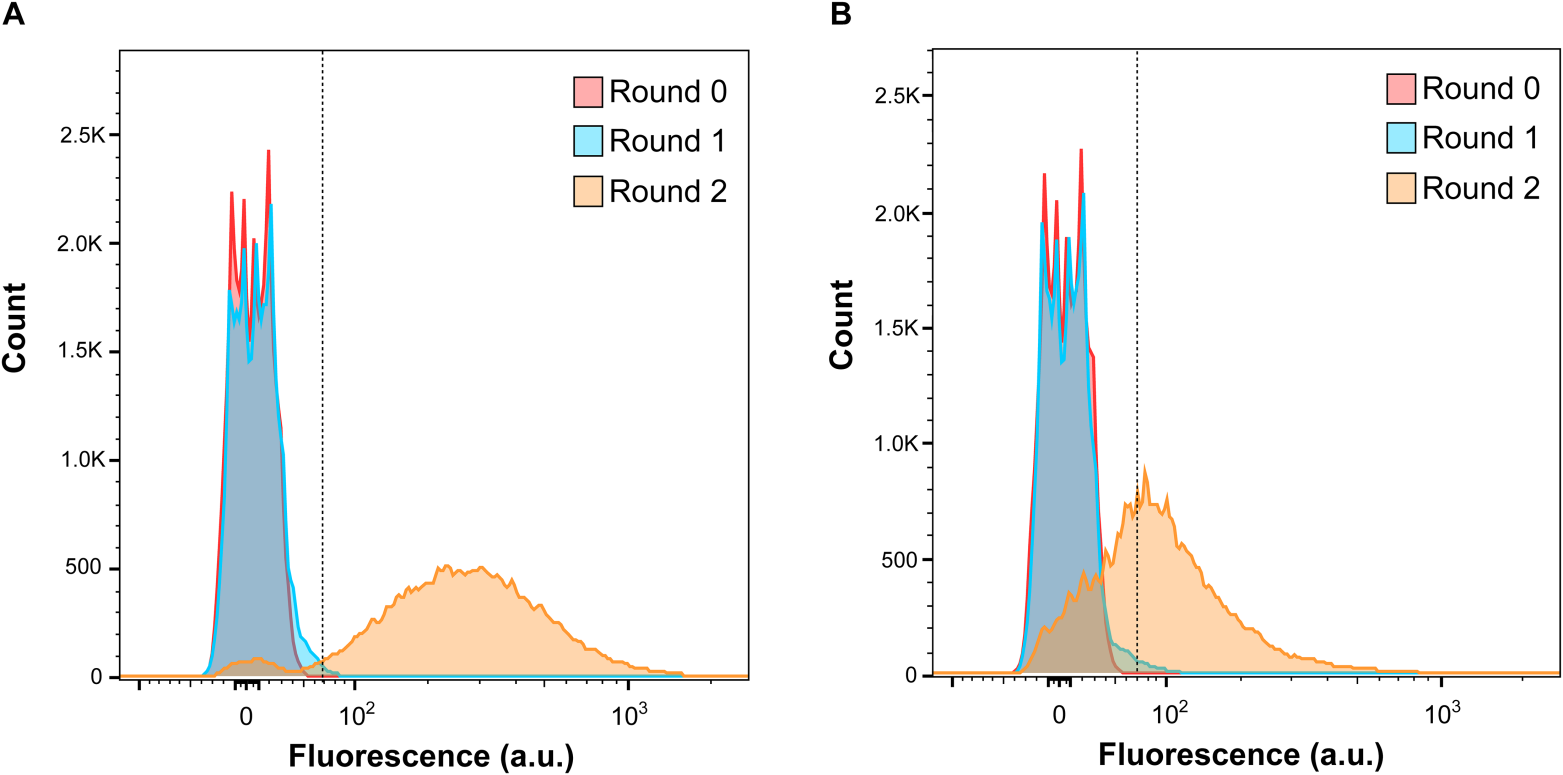
The RiboFACS approach facilitates sensitive detection of rare *ΔbtuB* cells in heterogeneous samples. The sensitivity of this approach was investigated by assessing the recovery of rare *ΔbtuB* cells from spiked mixtures containing primarily wild-type (WT) cells. Prior to sorting, both strains, which carried the AdoCbl-responsive riboswitch-based sensor, were individually grown in media supplemented with CNCbl, which has previously been shown to inhibit reporter gene expression in WT but not *ΔbtuB* cells (see Fig 3). Then, heterogeneous samples A and B were prepared by mixing the grown *ΔbtuB* and WT cells at ratios of (a) 1:200,000 and (b) 1:1,000,000, respectively. Finally, samples A and B were individually subjected to two-rounds of fluorescence activated cell sorting (FACS) using sort-gate P4 (as described in Fig 4): in the first-round, all of the cells within the unsorted samples (shown in red) were screened for AdoCbl-specific mutants; targeted cells were collected and regrown in the same media as above to yield the first-generation samples (shown in blue); afterwards, a second-round of cell sorting was performed on these first-generation samples from which between 20,000–50,000 cells were collected and regrown (shown in yellow). To track the enrichment progress, the fluorescence histograms corresponding to the unsorted and sorted populations of each artificial cell mixture sample were superimposed.

Having established a suitable gating strategy for the isolation of AdoCbl-specific mutants, the sensitivity of the approach was investigated by assessing the recovery of rare *ΔbtuB* cells (i.e. an AdoCbl-specific transport mutant) from spiked samples containing primarily WT cells. To prepare these artificial samples, initially, both *ΔbtuB* and WT cultures, carrying the AdoCbl-Rb-sfGFP sensor, were individually grown in media supplemented with CNCbl, which inhibits reporter fluorescence in WT but not *ΔbtuB* cells (see Fig 3). Next, samples ‘A’ and ‘B’ were prepared by mixing *ΔbtuB* and WT cells at ratios of 1:200,000 and 1:1,000,000, respectively (see the end of this section for the mathematical justification of these ratios). Anticipating that the detection of such low numbers of *ΔbtuB* cells within the “haystack” of WT cells would be challenging, each sample was subjected to two-rounds of sorting. In the first-round, all of the cells in the unsorted samples were screened using the previously established gating strategy via a “yield mask” setting (i.e. high yield, but low-accuracy stringency). Targeted cells were collected and regrown in the same media as above to be enriched prior to the next sort round, and they were subsequently considered, ‘first-generation’ samples. Afterwards, a second-round of cell sorting was performed on these first-generation samples, but via a “purity mask” setting (i.e. high-accuracy, but low-yield stringency). Finally, as a qualitative assessment of *ΔbtuB* cell enrichment, the fluorescence histograms corresponding to the unsorted and sorted populations of each artificial cell mixture sample were superimposed (Fig 5).

As shown in Fig 5, the right-sided shifts in the fluorescence histograms of the sorted populations relative to their unsorted counterparts suggest an enrichment of *ΔbtuB* cells by the RiboFACS approach. These mutants were originally present in the artificial cell mixture samples at very low quantities relative to WT cells. Prior to FACS (i.e. at round 0), the cells within samples A (Fig 5A) and B (Fig 5B) exhibited lack of reporter fluorescence. Expectedly, the fluorescence histograms of these populations were indistinguishable from that of a similarly grown WT population (S4 Fig). However, in as few as two rounds of FACS, the fluorescence histograms of both samples were considerably separated from their unsorted counterparts, but more so for sample A than for sample B. This was expected, as we hypothesized that these histogram shifts were the result of *ΔbtuB* cell enrichment (see below) and based on the fact that sample A originally contained five times the number of *ΔbtuB* cells as that present in sample B.

To confirm that the observed histogram shifts were in fact the result of *ΔbtuB* cell enrichment, cells were randomly selected from both twice-sorted samples, and they were screened by colony-PCR using *btuB* spanning primers and sequencing. The results confirmed our hypothesis, as all of the cells assayed from sample A and an even number of cells assayed from sample B, contained the *ΔbtuB* genotype. Notably, the observed proportions of *ΔbtuB* cells in both samples, A and B, conformed to their respective fluorescence histogram shifts relative to the minimum fluorescence intensity threshold of the sort gate. As shown in Fig 5A, the fluorescence histogram of twice-sorted sample A (yellow) showed that the vast majority of its cells fell within the sort gate and therefore were expected to present the *ΔbtuB* genotype. This was confirmed by our PCR and sequencing results. The fluorescence histogram of twice-sorted sample B (yellow), however, showed that an approximately even number of its cells fell either lower or higher than this threshold and therefore were expected to contain an approximately even mixture of both WT and *ΔbtuB* cells. This, too, was confirmed by our PCR and sequencing results. Incidentally, sample B could be sorted one more round to achieve higher *ΔbtuB* cell purity, as that of sample A.

Altogether, these findings demonstrate that the sensitivity of the RiboFACS-based method was sufficient to isolate rare *ΔbtuB* cells from artificial cell mixture samples, which contained them originally at very low abundances relative to a majority of WT cells. In particular, we were able to enrich for *ΔbtuB* cells (i.e. ~50% of the FACS-sorted population) from a sample that contained them and WT cells at a ratio of 1:1,000,000 after only two rounds of sorting (Fig 5B). To achieve higher cell purity, however, another round of sorting would likely be required. Comparatively, as few as two rounds of sorting were sufficient to substantially enrich for *ΔbtuB* mutants (i.e. >~95% of the FACS-sorted population) from a sample that contained them and WT cells at a ratio of 1:200,000 (Fig 5A).

Ultimately, we intend to use this method to isolate AdoCbl-specific mutants from a randomly mutagenized library of *E*. *coli*. Therefore, the sensitivity of the method was evaluated for this purpose. As described elsewhere [56,57], assuming a random distribution of transposase-mediated insertions in the genome of *E*. *coli*, the probability *P* of detecting an insertion in a given gene is based on the formula: *P* = 1 –(1– (x/g))^n^, where x is the length of the gene (assuming an average size of 1000 bps per gene), g is the size of *E*. *coli* genome (~4.6x10^6^ bps), and n is the number of mutants in our library. As such, one would require a library size of ~100,000 mutants to have a 99.99% chance of having at least one transposon insertion in every single gene of *E*. *coli*. Having demonstrated the ability of our approach to successfully isolate rare *ΔbtuB* mutants from artificial cell mixture samples, which contained them at a frequency between 1:1,000,000–1:200,000 relative to background cells, indicates that the sensitivity of the approach should be sufficient for the intended ultimate goal.

### Specificity assessment

**Fig 6:**
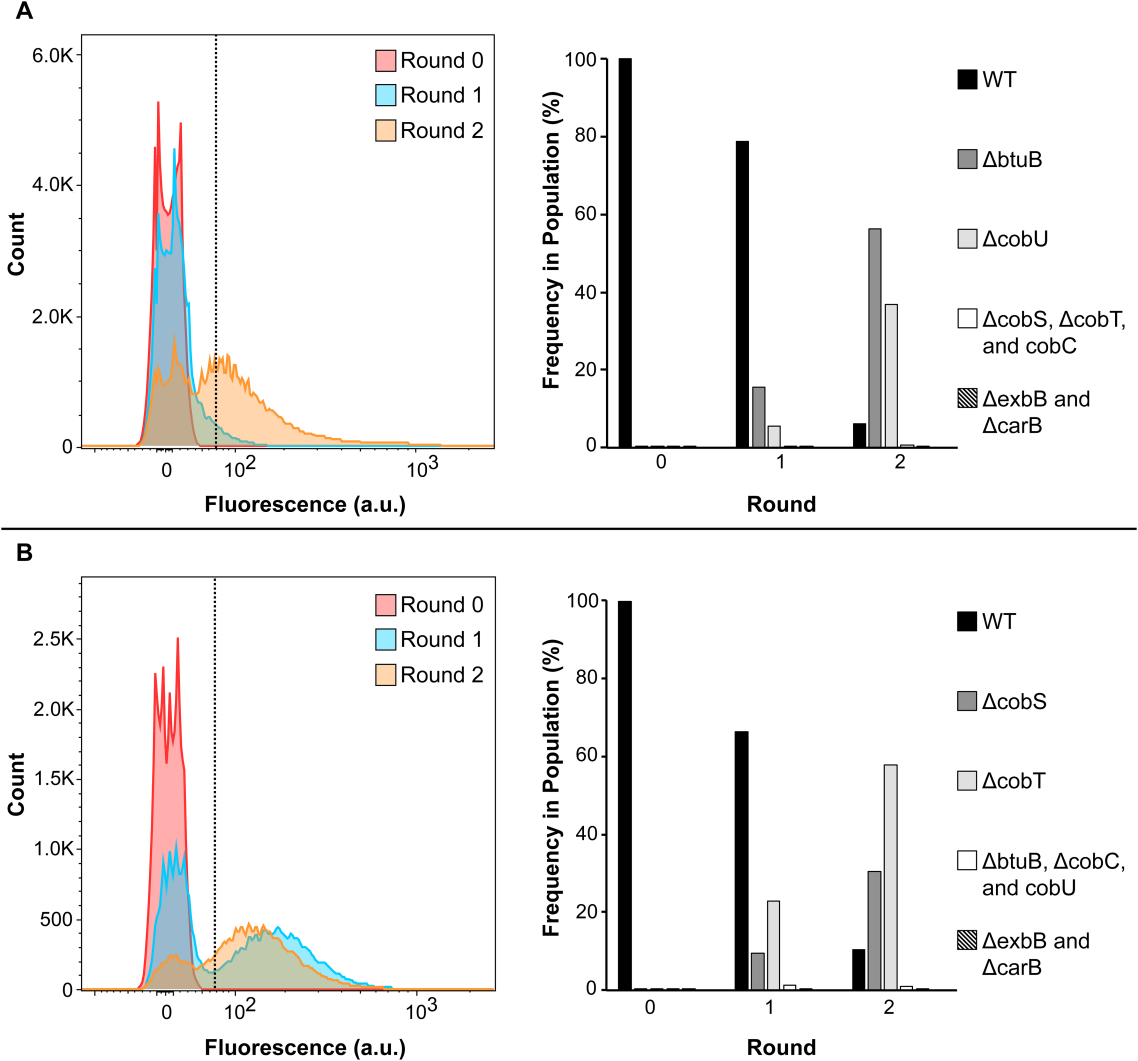
The RiboFACS approach facilitates the specific enrichment of mutants carrying disruptions in AdoCbl transport or biosynthesis. The specificity of RiboFACS was investigated by assessing the recovery of AdoCbl-specific mutants from spiked mixtures containing them (i.e. *ΔbtuB*, *ΔcobC*, *ΔcobU*, *ΔcobS*, *ΔcobT* and *ΔexbB*), as well as an unrelated mutant (*ΔcarB*), at extremely low quantities relative to a vast majority of wild-type (WT) cells. Prior to sorting, these strains, which carried the AdoCbl-responsive riboswitch-based sensor, were individually grown in media supplemented with Cbi & DMB. Then, replicate samples C and D were prepared by mixing each genetic knockout and WT cells at ratios of 1:200,000. These replicate samples were individually subjected to two-rounds of fluorescence activated cell sorting (FACS) using sort-gate P4 (as described in Fig 4): in the first-round, all of the cells within the unsorted sample’s (shown in red) were screened for AdoCbl-specific mutants; targeted cells were collected and regrown in the same media as above to yield the first-generation sample’s (shown in blue); afterwards, a second-round of cell sorting was performed on these first-generation sample’s from which ~50,000 cells were collected and regrown (shown in orange). To track the enrichment progress of replicate samples (a) C and (b) D, the fluorescence histograms corresponding to their unsorted and sorted populations were superimposed. Finally, the relative frequencies of the strains within the unsorted and sorted populations of each replicate sample were determined via deep sequencing, and plotted as bar graphs.

The specificity of the RiboFACS-based method was examined by assessing the recovery of AdoCbl-specific mutants from spiked samples containing known mixtures of them (i.e. *ΔbtuB*, *ΔcobC*, *ΔcobU*, *ΔcobS*, *ΔcobT* and *ΔexbB*), an unrelated mutant (*ΔcarB*) and WT cells. The experimental approach used in this section was similar to what was done for the sensitivity assessment with slight modifications. Briefly, the indicated strains were individually grown in media supplemented with precursors Cbi & DMB, which, as previously described (see Fig 3), may or may not lead to the inhibition of reporter gene expression (and hence a reduction in relative fluorescence) depending on the functional state of the host’s AdoCbl transport and biosynthetic processes. Replicates of artificial cell mixtures, labeled as samples C and D, were then prepared by combining each knockout strain (i.e. *ΔbtuB*, *ΔcobC*, *ΔcobU*, *ΔcobS*, *ΔcobT*, *ΔexbB*, and *ΔcarB*) and WT cells at a ratio of 1:200,000. Finally, these samples were separately subjected to two-rounds of sorting using the previously established gating strategy to assess the specificity with which AdoCbl-specific mutants may be enriched. To track the progress of their enrichment, the fluorescence histograms corresponding to the unsorted and sorted populations of replicate samples C and D were superimposed, as shown in Fig 6A and 6B (left panels), respectively. Moreover, these populations were deep sequenced both to identify their composition (in terms of the types of strains that they contain) and to measure the change in relative frequencies of the strains throughout the course of sorting, as displayed in Fig 6A and 6B (right panels).

As suggested by the superimposed fluorescence histograms in Fig 6A and 6B (left panels), considerable degrees of AdoCbl-specific mutant enrichment were achieved from both samples C and D. Prior to FACS (i.e. at round 0), the cells within the unsorted samples exhibited lack of reporter fluorescence, suggesting that the original amounts of AdoCbl-specific mutants within these populations were negligible. In as few as two-rounds of FACS, however, the fluorescence histograms of both samples were considerably right-shifted relative to their unsorted counterparts, suggesting an increase in the proportions of AdoCbl-specific mutants within these populations. Indeed, these interpretations were confirmed by analysis of the next-generation sequencing data obtained from these samples (see below).

Following next-generation sequencing (see read counts), a relative abundance score was calculated for each strain within the unsorted and sorted populations, by normalizing strain-specific read counts to total number of reads for each data set. The results were presented as bar graphs for both artificial cell mixture samples (Fig 6A and 6B, right panels). Expectedly, both samples C and D were determined to contain primarily WT cells prior to FACS (i.e. at round 0). However, in as few as two rounds of sorting, the fraction of WT cells in these replicate samples decreased significantly from ~100% to less than ~18%, paralleled by proportional increases in AdoCbl-specific mutants: in replicate sample C (Fig 6A, right panel), both *ΔbtuB* (58%) and *ΔcobU* (40%) mutants made up the majority of cells in the enriched population, whereas in replicate sample D (Fig 6B, right panel), the majority was represented by another pair of AdoCbl-specific mutants, namely *ΔcobT* (58%) and *ΔcobS* (26%). Possible explanations for why different sets of AdoCbl-specific mutants were identified from these replicate samples include factors that can affect the probability of their selection and enrichment: (1) the likely fact that the true numbers of each knockout strain in the two artificial cell mixture samples were not identical; and (2) the fact that the knockout strains do not exhibit the same degree of fluorescence intensities in their respective populations relative to the sort-gate’s fluorescence threshold. Nonetheless, both samples were enriched for only AdoCbl-specific mutants.

Strikingly, these results allude to the important roles that these genes are known to play in the transport and biosynthesis of AdoCbl in *E*. *coli* [7–9]. Deletion of any of these genes should result in lack of transport or biosynthesis of AdoCbl, hence uninhibited reporter gene expression, leading to fluorescence in their respective deletion strains, allowing for their enrichment by the RiboFACS approach and subsequent identification by next-generation sequencing.

Despite the increase of the indicated AdoCbl-specific mutants, no increase in *ΔcobC*, *ΔexbB* or *ΔcarB* cells were observed in either sample C or D. This is in agreement with the function of these genes and how they relate to AdoCbl uptake and biosynthesis, as described below.

We previously offered two explanations as to why we detected some fluorescence inhibition in a *ΔcobC* strain, in spite of it being an AdoCbl-specific mutant. These explanations related to the role of CobC in AdoCbl biosynthesis or to the characteristics of the riboswitch-based sensor itself. In either case, the resulting fluorescence behavior would lead to inefficient FACS-based isolation of *ΔcobC* mutants, as compared to the other AdoCbl-specific mutants.

ExbB is normally involved with ExbD in a protein complex that provides the energy transduction required for the uptake of vitamin B12 across the outer membrane [20]. However, as a previous report has shown, the function of ExbB and ExbD could be partially replaced by their homologs TolQ and TolR in *E*. *coli* [19,54,55]. This justifies the statistically significant reduction in the relative fluorescence intensity observed in *ΔexbB* (see Fig 3). Unsurprisingly, this would lead to inefficient FACS-based isolation of *ΔexbB* mutants, as compared to the other AdoCbl-specific mutants.

Lastly, *ΔcarB* is an example of a mutant unrelated to the transport and biosynthesis of AdoCbl [50]. As such, the AdoCbl-Rb-sfGFP sensor in this strain should behave similarly to that in the WT strain (i.e. exhibit inhibited fluorescence). Therefore, like WT cells, *ΔcarB* mutants would not be enriched by the RiboFACS-based approach, as was observed.

## Conclusion

We have previously demonstrated the effectiveness of the *E*. *coli* AdoCbl-responsive riboswitch sensor as an intracellular tool for monitoring the physiologically relevant concentrations of its cognate molecule. Herein, we examined the usefulness of this sensor in combination with FACS and next-generation sequencing to screen for cells with genetic disruptions in their AdoCbl transport and biosynthetic pathways (i.e. AdoCbl-specific mutants) among cells of other genotypes in artificial cell mixture samples. We show that this approach, dubbed RiboFACSeq, is able to isolate and identify AdoCbl-specific mutants with desirable sensitivity and specificity. Based on the success of our approach with artificial cell mixture samples, we believe that this approach can be applied to carry out the systematic elucidation of bacterial transport and biosynthesis pathways involved in the uptake and production of a metabolite of interest, as long as a suitable riboswitch-based sensor exists or can be created. This approach will be especially useful for the investigation of pathways that yield lowly abundant molecules, as their identification and separation through conventional means typically suffer from significant technical challenges.

## Supporting information

S1 Fig The structure, transport and biosynthesis of AdoCbl in *E*. *coli*. (A) Chemical structure of AdoCbl, a “complete” corrinoid. (B) Schematic of corrinoid import in *E*. *coli*. The salvaging of exogenous corrinoids (e.g. Cbi) is initiated by the outer-membrane transport protein BtuB, which binds to vitamin B12 (i.e. CNCbl) and a wide range of structural analogs. BtuB-mediated import into the periplasm requires energy input, which is acquired through interactions with an inner membrane protein complex comprised of proteins TonB, ExbB and ExbD. Specifically, in the presence of extracellular corrinoids, the Ton-box of BtuB unfolds and extends into the periplasm in order to initiate interaction with TonB, which provides energy in the form of PMF. Once in the periplasm, Cbi is bound by BtuF, which delivers it to the inner-membrane ABC transporter BtuCD. BtuCD translocates Cbi into the cytoplasm using ATP hydrolysis. The stoichiometry of the active TonB/ExbB/ExbD complex is speculative. (C) Schematic of the *E*. *coli* AdoCbl biosynthetic pathway from the incomplete corrinoid Cbi and the lower ligand base DMB. The first step in this pathway is the attachment of the upper axial ligand. Cbi is adenosylated by the ATP:corrinoid adenosyltransferase BtuR, which yields AdoCbi. Next, AdoCbi is converted into AdoCbi-GDP via an AdoCbi-P intermediate in a two-step process catalyzed by the bifunctional enzyme CobU, which has kinase and guanylyltransferase activities. Concurrently, the lower ligand base DMB is stimulated by the NaMN:DMB phosphoribosyl transferase enzyme CobT, which produces α-RP. Then, in the penultimate step, the AdoCbl-5’-P synthase enzyme CobS catalyzes the condensation of AdoCbi-GDP and α-RP to yield AdoCbl-5’-P. The last step in this biosynthetic pathway is catalyzed by the AdoCbl-P phosphatase enzyme CobC, which removes the 5’-O-P from AdoCbl-5’-P to form the end-product AdoCbl. Cbi, cobinamide; AdoCbi, adenosylcobinamide; AdoCbi-P, adenosylcobinamide-phosphate; AdoCbi-GDP, adenosylcobinamide guanosine diphosphate; AdoCbl-5’-P, adenosylcobalamin-5’-phosphate; AdoCbl, adenosylcobalamin; CNCbl, cyanocobalamin—also known as vitamin B12; DMB, 5,6-dimethylbenzimidazole; NaMN, nicotinate mononucleotide; α-RP, α-ribazole-5’-phosphate; ATP, adenosine triphosphate; NTP, nucleoside triphosphate; GTP, guanosine triphosphate; Pi, inorganic phosphate; PMF, protonmotive force.
(TIF)

S2 Fig Using the AdoCbl-Rb-sfGFP sensor to detect AdoCbl transport and metabolism in the strains used in this study. Initially, overnight cultures of each strain were prepared by growing cells in a rich, chemically defined medium (RDM) lacking vitamin B12 or its precursors. Afterwards, the overnights were resuspended (at 1:1000 dilution) in RDM supplemented with the following compounds: (1) neither CNCbl nor Cbi nor DMB (i.e. “no B12”); (2) CNCbl; or (3) Cbi and DMB. These cultures were grown until they reached ~mid-late log phase. Finally, the TECAN M1000 (Safire) plate-reader was used to read sfGFP fluorescence (488/509 nm). Each sample was assayed in triplicate, and its standard deviation was reported as error bars. A two-way ANOVA (with Bonferroni corrections) was run to determine the statistically significant differences between the samples (*, p-value < 0.05; **, p-value < 0.01; ***, p-value < 0.001; n.s., not significant).
(TIF)

S3 Fig The extent of VB12-mediated fluorescence fold-inhibition in the strains of this study. The ability of each strain to transport and synthesize AdoCbl was examined by measuring the reporter activities of cells grown in media supplemented with the following compounds: (i) no cyanocobalamin (CNCbl) nor cobinamide (Cbi) nor 5,6-dimethylbenzimidazole (DMB); (ii) CNCbl; and (iii) Cbi & DMB. Subsequently, the raw reporter activities were corrected for growth differences (OD600-normalized), and then used to determine the extent of fluorescence signal-inhibition in response to the indicated compound(s) relative to their absence. In other words, fluorescence fold-inhibition was calculated by dividing the fluorescence intensities in cells grown in the absence to that in the presence of CNCbl (orange) or both Cbi & DMB (purple), respectively. The lack of signal-inhibition, on the other hand, is defined by having a ratio of 1 (dashed line) or lower. Each bar represents the average of three biological replicates with errors as standard deviations. A two-way ANOVA (with Bonferroni corrections) was run to determine the statistically significant differences between the samples (*, p-value < 0.05; **, p-value < 0.01; ***, p-value < 0.001; n.s., not significant).
(TIF)

S4 Fig Fluorescence histogram comparison of WT and unsorted samples A and B. Initially, a WT cell culture (green) and samples A (orange) and B (blue), containing mixtures of *ΔbtuB* and WT cells at ratios of 1:200,000 and 1:1,000,000, respectively, were separately grown in a rich, chemically defined medium supplemented with vitamin B12 (CNCbl). Subsequently, the fluorescence histograms of these samples were acquired and superimposed.
(TIF)

S1 Table Strain-specific genetic barcodes. The table presents the genetic barcode of each strain used in this study.
(PDF)

S1 File Fluorescence behavior of AdoCbl-responsive riboswitch-based sfGFP sensor. Data corresponding to the fluorimetry-based detection of sfGFP fluorescence intensities in WT and mutant cells that were grown in a rich, chemically defined medium supplemented with or without vitamin B12 (CNCbl) or its precursors (Cbi & DMB). Accompanying the data set are the statistical outputs of running two-way ANOVAs on the indicated datasets.
(XLSX)

S2 File Flow cytometry data of WT and *ΔbtuB* cells grown with vitamin B12 (CNCbl). Data corresponding to the flow analysis of WT (WT_CNCbl.fcs) and *ΔbtuB* (btuB_KO_CNCbl.fcs) cells that were grown in a rich, chemically defined medium supplemented with vitamin B12 (CNCbl).
(ZIP)

S3 File Flow cytometry data of RiboFACS sensitivity assay. Data corresponding to the flow analysis of samples A and B, which originally contained mixtures of *ΔbtuB* and WT cells at ratios of 1:200,000 and 1:1,000,000, respectively, prior to (i.e. “PreSort”) and after FACS (i.e. “PostSort1” and “PostSort2”). Cells were grown and collected in a rich, chemically defined medium supplemented with vitamin B12 (CNCbl).
(ZIP)

S4 File Flow cytometry data of RiboFACS specificity assay. Data corresponding to the flow analysis of replicate samples C and D, which originally contained mixtures of all of the knockout strains used in this study (*ΔbtuB*, *ΔcobU*, *ΔcobS*, *ΔcobT*, *ΔcobC*, *ΔexbB*, and *ΔcarB*) at a ratio of 1:200,000 to WT cells, prior to (i.e. “PreSort”) and after FACS (i.e. “PostSort1” and “PostSort2”). Cells were grown and collected in a rich, chemically defined medium supplemented with vitamin B12 precursors Cbi & DMB.
(ZIP)
