# Supplementary figures and images for: RiboFACSeq: A new method for investigating metabolic and transport pathways in bacterial cells by combining a riboswitch-based sensor, fluorescence-activated cell sorting and next-generation sequencing
Source: PLoS One. 2017 Dec 6;12(12):e0188399. doi: 10.1371/journal.pone.0188399 (PMC5718407; doi:10.1371/journal.pone.0188399)

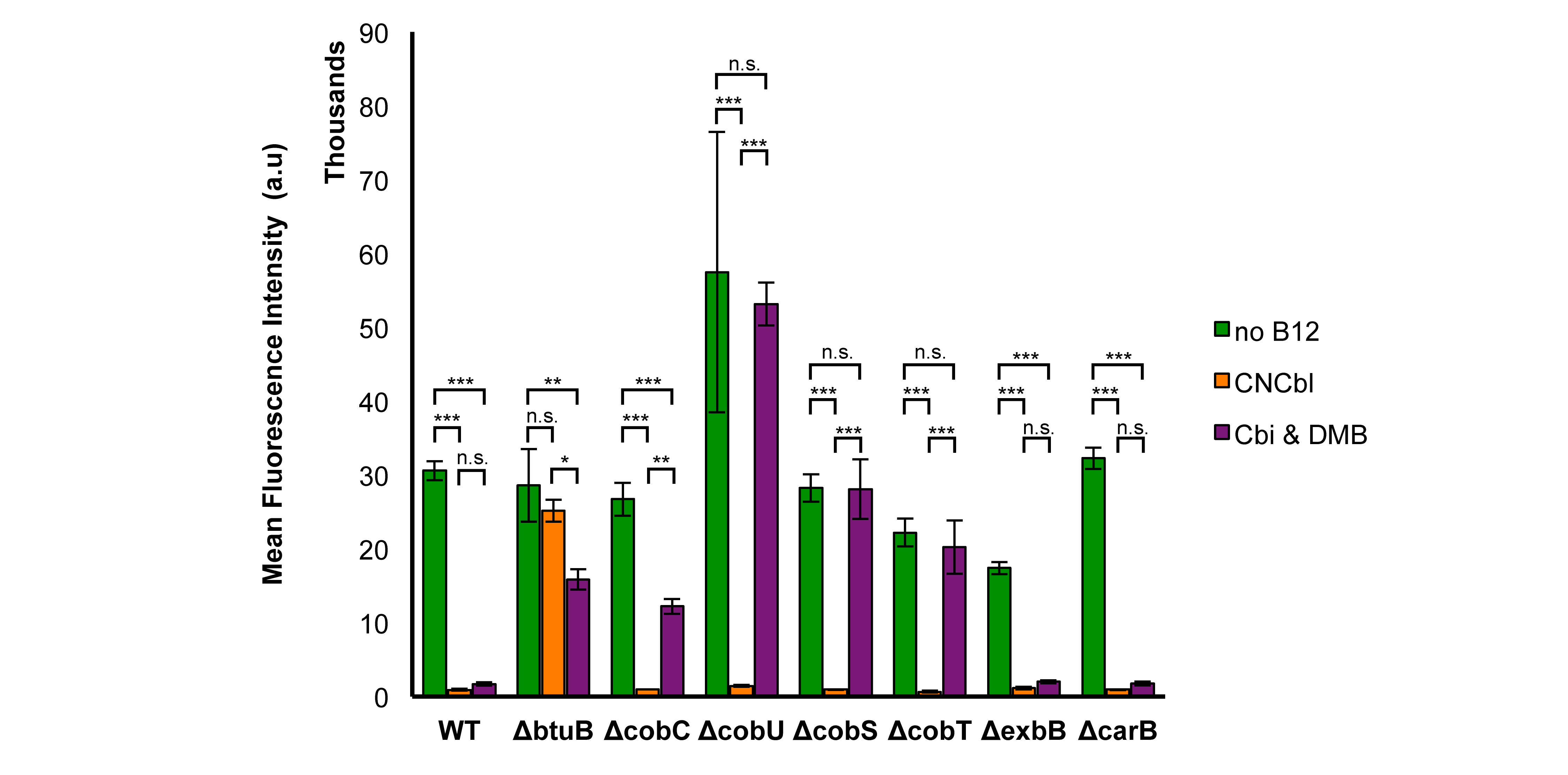

Supplement: S2 Fig — Initially, overnight cultures of each strain were prepared by growing cells in a rich, chemically defined medium (RDM) lacking vitamin B12 or its precursors. Afterwards, the overnights were resuspended (at 1:1000 dilution) in RDM supplemented with the following compounds: (1) neither CNCbl nor Cbi nor DMB (i.e. “no B12”); (2) CNCbl; or (3) Cbi and DMB. These cultures were grown until they reached ~mid-late log phase. Finally, the TECAN M1000 (Safire) plate-reader was used to read sfGFP fluorescence (488/509 nm). Each sample was assayed in triplicate, and its standard deviation was reported as error bars. A two-way ANOVA (with Bonferroni corrections) was run to determine the statistically significant differences between the samples (*, p-value < 0.05; **, p-value < 0.01; ***, p-value < 0.001; n.s., not significant). (TIF) [file pone.0188399.s002.tif]

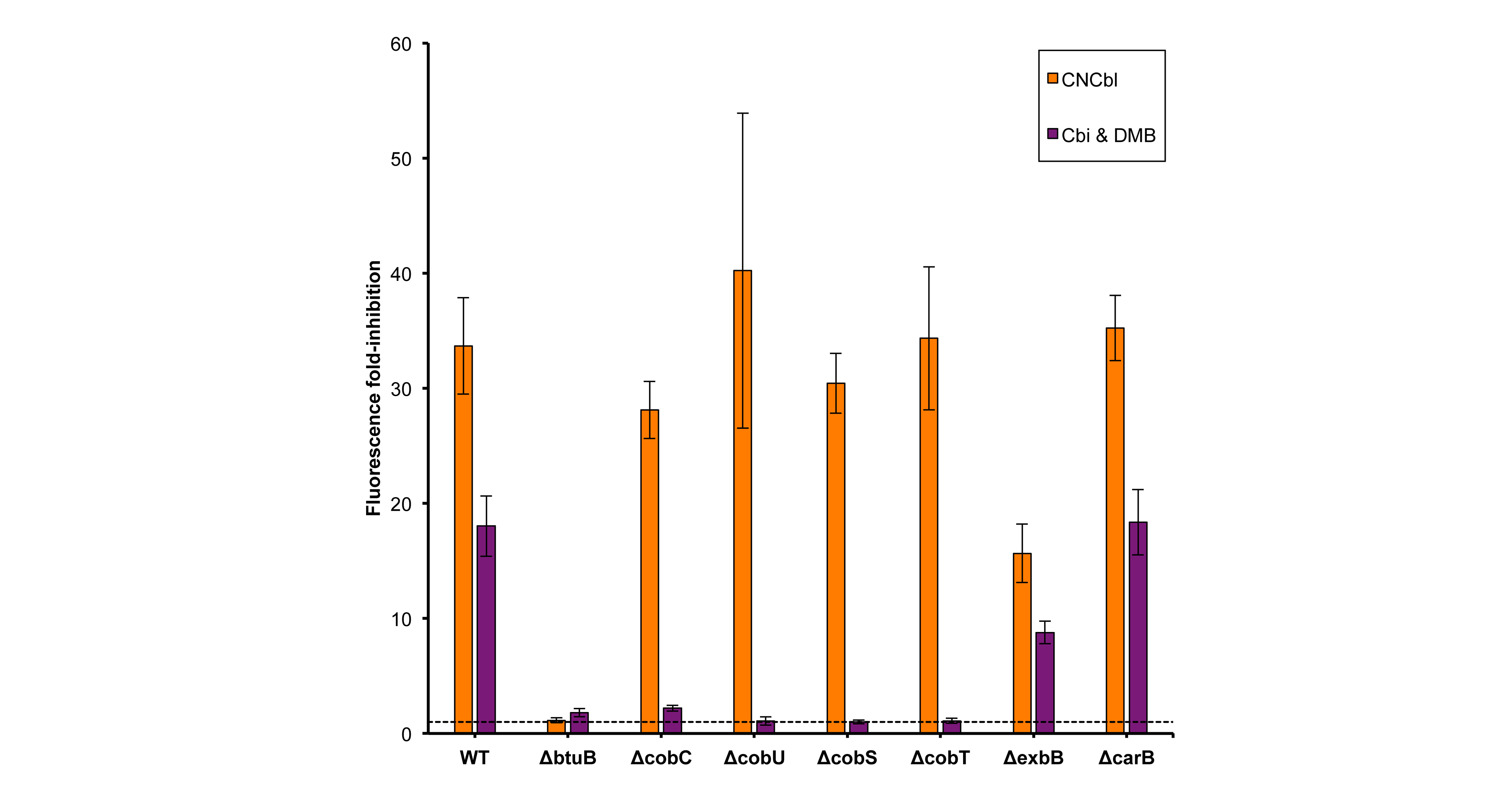

Supplement: S3 Fig — The ability of each strain to transport and synthesize AdoCbl was examined by measuring the reporter activities of cells grown in media supplemented with the following compounds: (i) no cyanocobalamin (CNCbl) nor cobinamide (Cbi) nor 5,6-dimethylbenzimidazole (DMB); (ii) CNCbl; and (iii) Cbi & DMB. Subsequently, the raw reporter activities were corrected for growth differences (OD600-normalized), and then used to determine the extent of fluorescence signal-inhibition in response to the indicated compound(s) relative to their absence. In other words, fluorescence fold-inhibition was calculated by dividing the fluorescence intensities in cells grown in the absence to that in the presence of CNCbl (orange) or both Cbi & DMB (purple), respectively. The lack of signal-inhibition, on the other hand, is defined by having a ratio of 1 (dashed line) or lower. Each bar represents the average of three biological replicates with errors as standard deviations. A two-way ANOVA (with Bonferroni corrections) was run to determine the statistically significant differences between the samples (*, p-value < 0.05; **, p-value < 0.01; ***, p-value < 0.001; n.s., not significant). (TIF) [file pone.0188399.s003.tif]

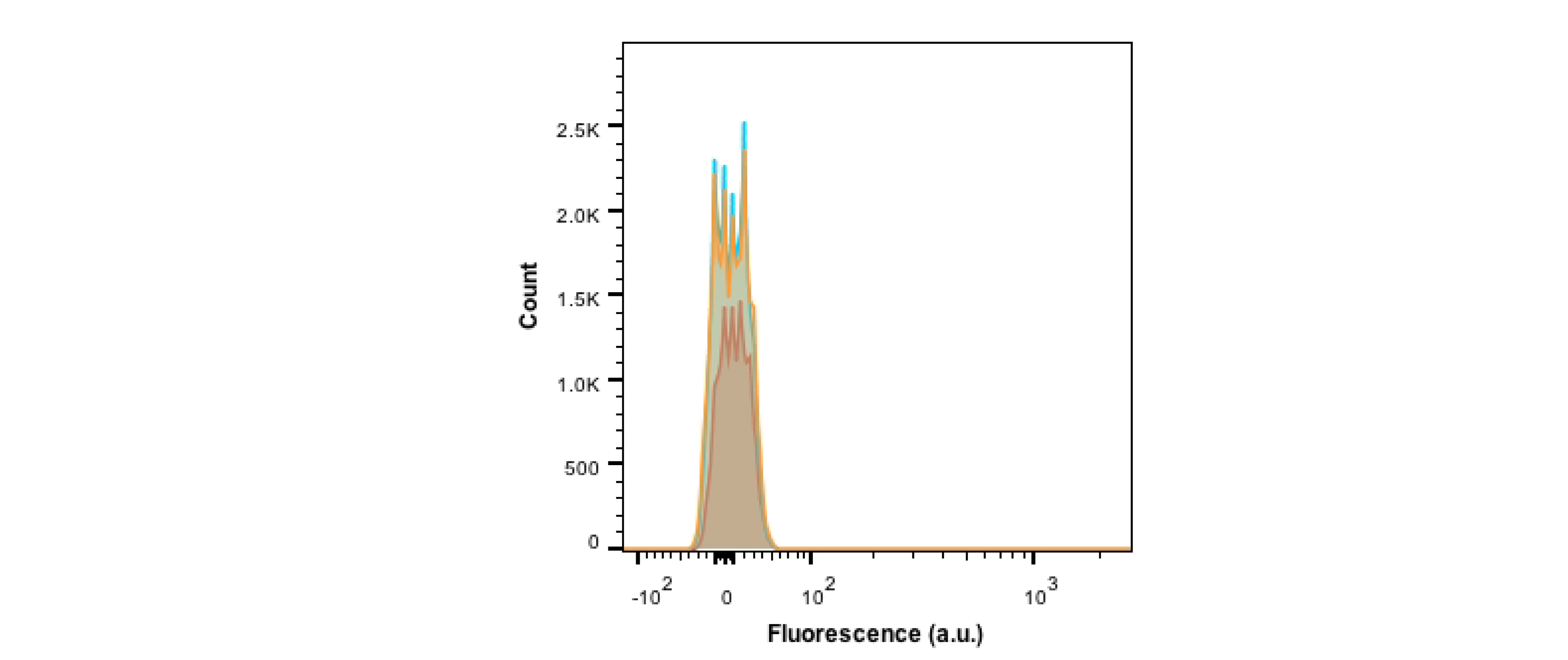

Supplement: S4 Fig — Initially, a WT cell culture (green) and samples A (orange) and B (blue), containing mixtures of ΔbtuB and WT cells at ratios of 1:200,000 and 1:1,000,000, respectively, were separately grown in a rich, chemically defined medium supplemented with vitamin B12 (CNCbl). Subsequently, the fluorescence histograms of these samples were acquired and superimposed. (TIF) [file pone.0188399.s004.tif]
