# Supplementary material for: RiboFACSeq: A new method for investigating metabolic and transport pathways in bacterial cells by combining a riboswitch-based sensor, fluorescence-activated cell sorting and next-generation sequencing
Source: PLoS One. 2017 Dec 6;12(12):e0188399. doi: 10.1371/journal.pone.0188399 (PMC5718407; doi:10.1371/journal.pone.0188399)
Supplement: S1 Table — The table presents the genetic barcode of each strain used in this study. (PDF) [file pone.0188399.s005.pdf]

**Strain**	**Genetic ID**
WT	ATGTGTGTA
ΔbtuB	ATGGCTTGT
ΔcobC	ATGATCTGT
ΔcobU	ATGCTGTGT
ΔcobS	ATGTTCTGT
ΔcobT	ATGCCTTGT
ΔexbB	ATGGTATGT
ΔcarB	ATGTGTTGT
